# Colony-Stimulating Factor-1 Receptor Inhibition Transiently Attenuated the Peripheral Immune Response to Experimental Traumatic Brain Injury

**DOI:** 10.1089/neur.2022.0092

**Published:** 2023-04-28

**Authors:** Katherine R. Giordano, Maha Saber, Tabitha R.F. Green, Luisa M. Rojas-Valencia, J. Bryce Ortiz, Sean M. Murphy, Jonathan Lifshitz, Rachel K. Rowe

**Affiliations:** ^1^ BARROW Neurological Institute at Phoenix Children's Hospital, Phoenix, Arizona, USA.; ^2^ Department of Child Health, University of Arizona College of Medicine–Phoenix, Phoenix, Arizona, USA.; ^3^ Phoenix Veteran Affairs Health Care System, Phoenix, Arizona, USA.; ^4^ Department of Integrative Physiology, University of Colorado Boulder, Boulder, Colorado, USA.

**Keywords:** concussion, inflammation, microglia, peripheral immune response, PLX

## Abstract

To investigate microglial mechanisms in central and peripheral inflammation after experimental traumatic brain injury (TBI), we inhibited the colony-stimulating factor-1 receptor (CSF-1R) with PLX5622 (PLX). We hypothesized that microglia depletion would attenuate central inflammation acutely with no effect on peripheral inflammation. After randomization, male mice (*n* = 105) were fed PLX or control diets (21 days) and then received midline fluid percussion injury or sham injury. Brain and blood were collected at 1, 3, or 7 days post-injury (DPI). Immune cell populations were quantified in the brain and blood by flow cytometry. Cytokines (interleukin [IL]-6, IL-1β, tumor necrosis factor-α, interferon-γ, IL-17A, and IL-10) were quantified in the blood using a multi-plex enzyme-linked immunosorbent assay. Data were analyzed using Bayesian multi-variate, multi-level models. PLX depleted microglia at all time points and reduced neutrophils in the brain at 7 DPI. PLX also depleted CD115^+^ monocytes, reduced myeloid cells, neutrophils, and Ly6C^low^ monocytes in blood, and elevated IL-6. TBI induced a central and peripheral immune response. TBI elevated leukocytes, microglia, and macrophages in the brain and elevated peripheral myeloid cells, neutrophils, Ly6C^int^ monocytes, and IL-1β in the blood. TBI lowered peripheral CD115^+^ and Ly6C^low^ monocytes in the blood. TBI PLX mice had fewer leukocytes and microglia in the brain at 1 DPI, with elevated neutrophils at 7 DPI compared to TBI mice on a control diet. TBI PLX mice also had fewer peripheral myeloid cells, CD115^+^, and Ly6C^low^ monocytes in the blood at 3 DPI, but elevated Ly6C^high^, Ly6C^int^, and CD115^+^ monocyte populations at 7 DPI, compared to TBI mice on a control diet. TBI PLX mice had elevated proinflammatory cytokines and lower anti-inflammatory cytokines in the blood at 7 DPI compared to TBI mice on a control diet. CSF-1R inhibition reduced the immune response to TBI at 1 and 3 DPI, but elevated peripheral inflammation at 7 DPI.

## Introduction

Traumatic brain injury (TBI) is a leading cause of death and disability in the world and affects persons across the life span.^1^ TBI is induced by a mechanical impact that triggers pathophysiological processes, which include both central and peripheral inflammation. The time course and magnitude of inflammation after TBI can be highly variable because of multiple factors that include injury type, impact location, injury severity, individual age, and access to care. Despite the variability across cases, post-traumatic inflammation is critical in the pathophysiology and recovery from TBI and can have detrimental effects if unregulated.^2–4^

In response to TBI, immune cells of the central nervous system, including resident microglia, undergo morphological changes and begin producing pro- and anti-inflammatory cytokines and other signaling molecules.^5^ Blood–brain barrier damage and cytokine signaling from central immune cells leads to infiltration of neutrophils and monocyte-derived macrophages into the parenchyma and continued cytokine signaling, which perpetuates immune cell activation, creating a feedforward loop.^6,7^ Acutely after injury, microglia and monocyte-derived macrophages remove cellular debris and promote tissue remodeling through synaptic stripping and trophic support.^8^ However, microglia and monocyte-derived macrophages can release neurotoxic substances that can exacerbate injury and worsen clinical symptoms.^9,10^

In addition to central inflammation, TBI can elicit a peripheral immune response.^11–13^ Inflammatory signaling molecules released into the blood recruit peripheral immune cells to the brain and increase peripheral monocyte numbers in the circulation.^13,14^ Downstream consequences of increased circulatory inflammation after TBI include immune cell infiltration into peripheral organs, which can lead to organ dysfunction and adverse outcomes.^11,12^

Anti-inflammatory therapeutics have yet to be successful after TBI, but remain plausible targets for intervention because of the dual role of neuroinflammation in both injury and recovery.^2,15^ It is critical to further understand the mechanisms of both central and peripheral TBI-induced inflammation in order to identify effective therapeutics. Microglia are essential in both acute and chronic inflammation in the brain after TBI, and therefore targeting microglial activity has been an important approach to elucidate the neuroinflammatory mechanisms. Colony-stimulating factor-1 receptor (CSF-1R) is critical for microglial survival, and the inhibition of CSF-1R through genetic knockouts or pharmacological treatment depletes brain microglia.^16^ Although complete microglia depletion may not be a practical therapy for persons after TBI, microglia depletion as a pharmacological research tool can lend valuable insight into the microglial mechanisms of TBI-induced inflammation. Oral administration of the CSF-1R inhibitor, PLX5622 (PLX), rapidly depletes >95% of microglia in the brain within 7 days, and after removal from a PLX diet, microglia repopulate the brain.^17^

In this study, we used PLX to deplete microglia, without repopulation, and isolate the microglial mechanisms of both central and peripheral inflammation. We hypothesized that microglia depletion with PLX would attenuate central inflammation with no effect on peripheral inflammation, as evidenced by immune cell populations and inflammatory cytokine levels in the brain and blood.

## Methods

### Rigor

All animal studies were conducted in accordance with the guidelines established by the Institutional Animal Care and Use Committee at the University of Arizona and the National Institutes of Health guidelines for the care and use of laboratory animals. Studies are reported following the Animal Research: Reporting In Vivo Experiments (ARRIVE) guidelines.^18^ Animals were randomly assigned to treatment groups before initiation of the study to ensure an equal distribution of experimental conditions across groups. Data collection stopped at pre-determined end-points based on days post-injury (DPI) for each animal. Quantification of immune cell populations and cytokines was performed by investigators blind to the experimental treatments.

### Animals

Adult male C57BL/6J mice (20–25 g; The Jackson Laboratory, Bar Harbor, ME) were used for all experiments (*n* = 105). Mice were group-housed in a 14:10 light-dark cycle at a fixed temperature (23°C ± 2°C) with food (AIN-76A rodent chow; Research Diets, Inc., New Brunswick, NJ) and water available *ad libitum*, according to the Association for Assessment and Accreditation of Laboratory Animal Care International. Group sizes were as follows: control sham: 1 DPI, *n* = 9; control TBI: 1 DPI, *n* = 10; PLX sham: 1 DPI, *n* = 7; PLX TBI: 1 DPI, *n* = 8; control sham: 3 DPI, *n* = 9; control TBI: 3 DPI, *n* = 8; PLX sham: 3 DPI, *n* = 9; PLX TBI: 3 DPI, *n* = 10; control sham: 7 DPI, *n* = 8; control TBI: 7 DPI, *n* = 7; PLX sham: 7 DPI, *n* = 10; and PLX TBI: 7 DPI, *n* = 10.

### Plexxikon administration

After a 7-day acclimation period post-shipping, mice either remained on control AIN-76A rodent chow or were switched to PLX5622 (1200 mg/kg; PLX) formulated in AIN-76A rodent chow for 21 days before experimental procedures. Access to food and water remained *ad libitum*, and mice remained on control or PLX diets continuously until the end of the study.

### Midline fluid percussion injury

Mice were subjected to midline fluid percussion injury (mFPI), as previously described.^19,20^ Mice were anesthetized with 5% isoflurane in 100% oxygen for 3 min and placed into a stereotaxic frame with continuously delivered 2.5% isoflurane by nose cone. Body temperature was maintained using a Deltaphase isothermal heating pad (Braintree Scientific, Inc., Braintree, MA). A midline incision exposed the bregma and lambda. Fascia was removed from the surface of the skull, and a craniectomy was performed with a trephine (3-mm outer diameter) on the sagittal suture midway between the bregma and lambda without disruption of the dura. An injury cap was prepared from a Luer-Loc needle hub and fixed over the craniectomy using cyanoacrylate gel and methyl-methacrylate (Hygenic Corp., Akron, OH). The hub was filled with saline and closed with a cap made from a modified syringe tip to prevent debris and air exposure overnight. Animals were placed in a heated recovery cage and monitored until they were ambulatory before being placed back in their home cage.

Mice were reanesthetized 24 h after surgery with 5% isoflurane in 100% oxygen for 3 min. The cap was removed from the hub and the dura inspected to ensure that it was intact. The hub was filled with saline and attached to an extension tube connected to the fluid percussion injury (FPI) device (Custom Design and Fabrication; Virginia Commonwealth University, Richmond, VA). When a toe-pinch response was detected, a moderate injury (1.2–1.4 atm) was administered by releasing the pendulum onto the fluid-filled cylinder, which delivered a pulse directly onto the intact dura. Mice were immediately monitored for a positive fencing response and righting reflex recovery time.^21^ After mice had spontaneously righted, brains were inspected for uniform herniation, hematoma, and dura integrity. Sham animals were attached to the FPI device, but the pendulum was not released. Incisions were sutured and mice were placed in a heated recovery cage and monitored until they were ambulatory. Animal welfare was monitored and documented daily during physical examination post-operative care. Food and water consumption were monitored, and body weights were collected daily as part of the routine post-operative assessment.

### Tissue collection

At pre-determined time points post-injury (1, 3, or 7 DPI), brain and blood were collected. Approximately 300 μL of blood was collected by submandibular bleeds in BD Microtainer^®^ MAP microtubes coated in ethylenediaminetetraacetic acid (Becton, Dickinson, and Company, Franklin Lakes, NJ). Blood (100 μL) was immediately processed for flow cytometry. The remaining blood (∼200 μL) was centrifuged to collect plasma for each mouse. Plasma samples were stored at −80°C until cytokine quantification. Immediately after blood collection, mice were injected (intraperitoneally) with Euthasol (0.002 mL/g; Patterson Veterinary, Greeley, CO) and perfused with ice-cold 1 × phosphate-buffered saline. Brains were removed and hemisected. One entire hemisphere was immediately processed for flow cytometry.

### Flow cytometry

#### Brain

Brain hemispheres were digested with the MACS^®^ Neural Tissue Dissociation Kit (Miltenyi Biotec, Bergisch Gladbach, Germany). Myelin was removed from samples with 30% Percoll gradient (GE Healthcare, Chicago, IL). Next, samples were blocked with Fc block (1:1000; BioLegend, San Diego, CA) for 10 min and then incubated with the following antibodies for ∼15 min: CD45 (1:300; BioLegend); CD11b (1:300; BioLegend); Ly6G (1:500; BioLegend); and Ly6C (1:300; BioLegend). Samples were washed in fluorescence-activated cell sorting (FACS) buffer, and cell populations were sorted with a BD FACSCanto™ II Cell Analyzer (BD Biosciences, San Jose, CA). Cells were gated on CD11b^+^ events and separated by CD45^low^ versus CD45^high^ expression. CD45^+^ cells were further gated on Ly6C^int^Ly6G and Ly6C expression to determine the percentage of infiltrating neutrophils and monocytes, respectively. Macrophages were defined as CD11b^+^CD45^high^, and resident microglia were defined as CD11b^+^CD45^low^.

**FIG. 1.**
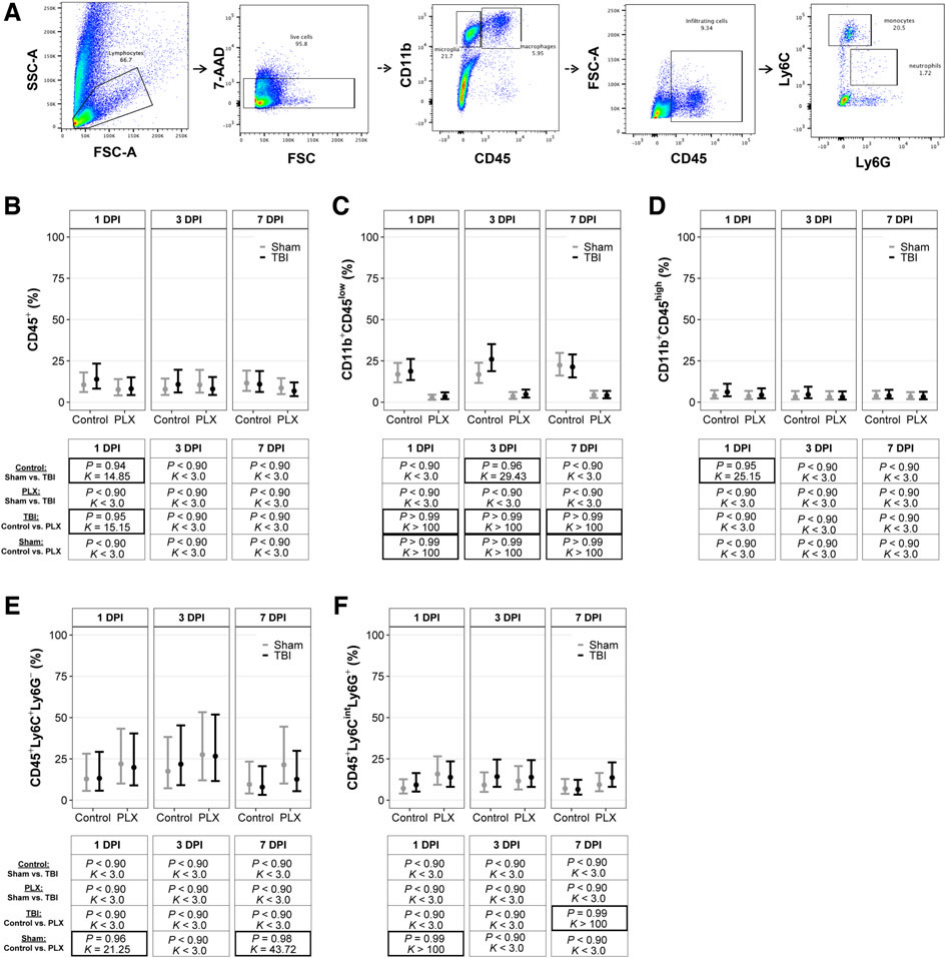
CSF-1R inhibition depleted microglia in the brain independent of TBI. (**A**) Flow cytometry gating strategies for brain samples. Immune cell populations in the brain for (**B**) leukocytes, (**C**) microglia, (**D**) macrophages, (**E**) monocytes, and (**F**) neutrophils. Results are presented graphically as marginal effects point estimates with 95% credible intervals along with tables of corresponding posterior probabilities (*P*) and Bayes factors (*K*). Statistically supported differences are indicated by bold black boxes. 7-AAD, 7-aminoactinomycin D; CSF-1R, colony-stimulating factor-1 receptor; DPI, days post-injury; FSC, forward scatter; FSC-A, forward-scatter area; PLX, PLX5622; SSC-A, side-scatter area; TBI, traumatic brain injury.

Cell populations are presented as the number of live events in Supplementary Table S1. The total number of events of interest for microglia and macrophage populations was taken as a ratio of live events to calculate the percentage of cells of interest, whereas neutrophils and monocyte populations were taken as a ratio to CD45^+^ cells to calculate the percentage of cells of interest. A diagram of the gating strategy is provided in Figure 1A.

#### Blood

**FIG. 2.**
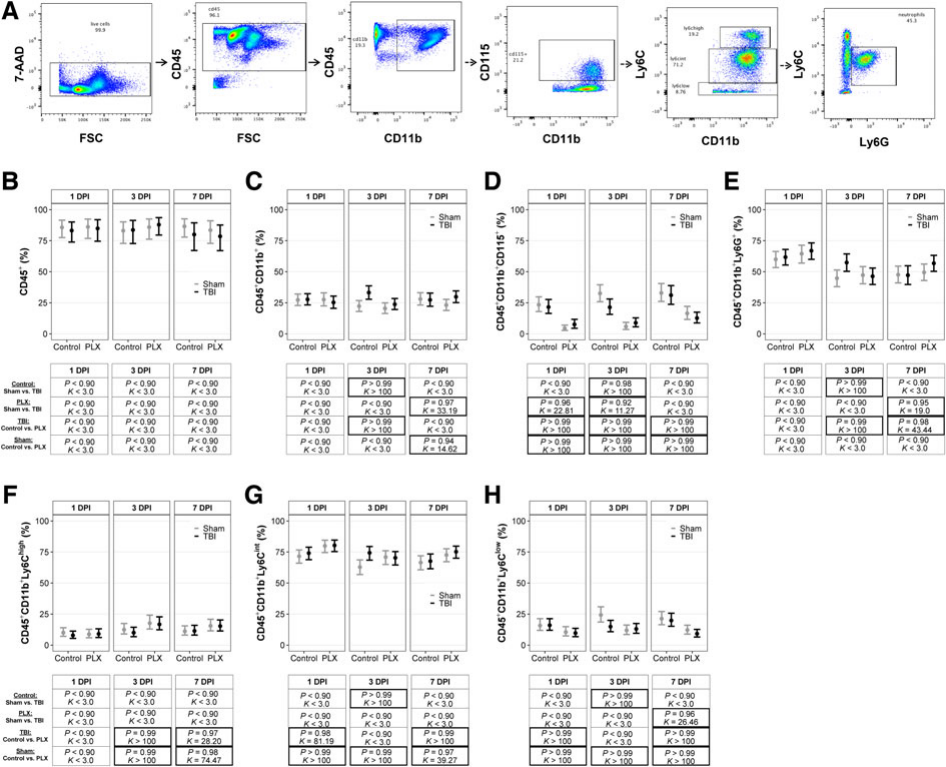
CSF-1R inhibition altered monocyte populations and prevented TBI-induced increases of peripheral myeloid cells and neutrophils. (**A**) Flow cytometry gating strategies for blood samples. Peripheral immune cell populations in the blood for (**B**) leukocytes, (**C**) CD115^+^ monocytes, (**D**) neutrophils, (**E**) monocytes, and (**F–H**) Ly6C^+^ monocytes. Results are presented graphically as marginal effects point estimates with 95% credible intervals along with tables of corresponding posterior probabilities (*P*) and Bayes factors (*K*). Statistically supported differences are indicated by bold black boxes. 7-AAD, 7-aminoactinomycin D; CSF-1R, colony-stimulating factor-1 receptor; DPI, days post-injury; FSC, forward scatter; FSC-A, forward-scatter area; PLX, PLX5622; SSC-A, side-scatter area; TBI, traumatic brain injury.

Whole-blood samples (100 μL) were incubated with Fc block (1:500; BioLegend) for 10 min and then incubated with the following antibodies for 15 min: CD45 (1:200; BioLegend); CD11b (1:200; BioLegend); Ly6C (1:100; BioLegend); Ly6G (1:200; BioLegend); CD115 (1:100; BioLegend); NK1.1 (1:100; BD BioSciences); B220 (1:200; BioLegend); and Siglec F (1:100; BD BioSciences). Next, samples were lysed with 1X Red Blood Cell Lysis Buffer (BioLegend) and washed with FACS buffer. Cell populations were sorted with a BD FACSCanto II Cell Analyzer (BD Biosciences). Briefly, 80,000 CD45^+^ events were collected. 7-aminoactinomycin D (7-AAD) was used to separate live and dead cells. CD45^+^ cells were then gated on CD11b^+^ events, then gated on Ly6C, Ly6G, and Cd115. To isolate monocyte and neutrophil populations, CD3, B220, Siglec F, and NK1.1 were used to remove T cells, B cells, eosinophils, and natural killer cells, respectively. Cell populations are presented as the number of live events in Supplementary Table S2. The total number of events of interest was taken as a ratio to CD11b^+^ cells to calculate the percentage of cells of interest. A diagram of the gating strategy is provided in Figure 2A.

### Cytokine quantification

Cytokine assays (Bio-Plex Pro™ Mouse Cytokine Th17 Panel A 6-Plex; Bio-Rad Laboratories, Hercules, CA) were performed according to manufacturer's instructions to quantify levels of interleukin (IL)-6, IL-1β, tumor necrosis factor (TNF)-α, interferon (IFN)-γ, IL-17A, and IL-10 in plasma. Briefly, samples were diluted with sample diluent at 1:4. Samples and supplied standards were transferred to a 96-well plate containing antibodies coupled to magnetic beads for each analyte and incubated on a shaker for 30 min at room temperature (RT). Samples were washed three times with supplied wash buffer and then incubated with detection antibodies on a shaker for 30 min at RT. Samples were washed three times with supplied wash buffer and then incubated with Streptavidin-PE on a shaker for 10 min at RT. Samples were washed three times with supplied wash buffer. Beads were resuspended for 5 min and then quantified on a Bio-Plex^®^ 200 system (Bio-Rad Laboratories). All samples were run in duplicate, and duplicate values were averaged.

### Statistical analyses

Similar to recent studies that analyzed cytokines and immune cell population flow data from experimental TBI,^22,23^ we conducted all statistical analyses using Bayesian multi-variate, multi-level models. We first subset the immune cell population data by the source from which they originated (i.e., blood or brain). We then fit multi-variate models in which each cytokine or immune cell population was analyzed simultaneously with other cytokine or immune cell populations from the same source or brain region. Thus, we fit a total of five multi-variate models, each with five to seven submodels that corresponded to each cytokine or immune cell population, for a total of 30 submodels. Population-level effects (*sensu* fixed effects in frequentist inference) in each model were the categorical variables diet, injury, and DPI, as well as a three-way interaction among those variables. We included group-level varying intercepts (*sensu* random effects)^24^ for cohorts to account for potential variation that may have been introduced by cohort-grouped entry into the study (e.g., each cohort of animals was injured on a different day, etc.).

Based on the findings of previous similar studies, cytokine data are typically best described by log-normal distributions, so we specified log-normal response distributions in each cytokine model.^22,23^ Compared to other approaches for analyzing log-normal data, such as transforming the data to be approximately normal and fitting models that assume normality, fitting log-normal models can result in unbiased parameter estimates and more accurate interval coverage.^25,26^ In contrast, immune cell population data are percentages bounded between zero and one, which are best described by beta distributions.^27,28^ Therefore, we specified beta response distributions for all immune cell population models, which is a superior approach for analyzing percentages, proportions, and ratios compared to applying methods that assume data are normally distributed, primarily because the normal distribution allows values to implausibly range from –∞ to ∞ rather than confined between zero and one.^29^ Additionally, we accommodated heteroscedasticity (i.e., unequal variances) between diet groups and injured groups by specifying variable-specific residual variance components in each model.^30^

We applied conservatively informative priors to all model parameters and variance components, based on results of previous studies,^22,23^ results from preliminary studies conducted by our group, and recommendations from past statistical research.^31^ Specifically, we applied ∼Normal(0, 1) priors to population-level parameters, ∼Cauchy(0, 5) priors to the scale parameters, and ∼Cauchy(0, 1) to the standard deviations of group-level effects.^31^ We fit models using the Stan computational platform^32^ implemented with the packages *rstan* and *brms* in the R statistical computing environment.^30,33,34^ We ran four Markov chains for each model, with a burn-in for each chain of 2000 iterations of the No-U-Turn Sampler extension to Hamiltonian Monte-Carlo sampling, followed by 3000 sampling iterations, which produced 12,000 total posterior samples for each model.

We assessed model convergence using trace plots and estimates of the potential scale reduction factor ($\hat{R}$) and effective sample sizes (*n*_eff_); optimal values for $\hat{R}$ and *n*_eff_ were strictly 1.00–1.01 and >1000, respectively.^35,36^ We assessed model fit using posterior predictive check plots created with the R package *bayesplot*, comparing 1000 posterior predictive distribution samples to the observed data.^37–39^ We based inferences on a combination of model parameter estimates (*θ*; posterior means), 95% credible intervals, and conditional marginal effects under the posterior distributions, as well as the estimated posterior probabilities (*P*) and Bayes factors (*K*;^40,41^) from multiple-comparisons tests conducted by non-linear hypothesis testing in the R package *brms*. We provide a summary table of the statistical metrics used to evaluate the strength of support for the results (Table 1).

**Table 1.** Statistical Metrics to Evaluate Strength of Support

	Strength of support			
Metric	Weak	Moderate	Strong	Decisive
Posterior probability (*P*)	0.90–0.92	0.93–0.95	0.96–0.98	≥0.99
Bayes factor (*K*)	<3	3–10	11–100	>100

## Results

### Colony-stimulating factor-1 receptor inhibition in traumatic brain injury mice lowered leukocytes 1 day post-injury and elevated neutrophils at 7 days post-injury

Immune cell populations were quantified using flow cytometry in brain tissue (Fig. 1A). All posterior probabilities (*P*) and Bayes factors (*K*) are presented with corresponding graphs of point estimates and confidence intervals in Figure 1. There was a TBI effect on leukocyte populations in the brain at 1 DPI. In mice on the control diet, there were more leukocytes in TBI mice compared to sham mice (Fig. 1B). There was also a PLX effect in TBI mice; TBI mice on the PLX diet had fewer leukocytes than TBI mice on the control diet. There were no differences in leukocyte populations in the brain at 3 or 7 DPI. Mice on the PLX diet had fewer microglia compared to mice on the control diet at 1, 3, and 7 DPI, independent of TBI (Fig. 1C). At 3 DPI, there was a TBI effect on microglia; TBI mice on the control diet had more microglia than sham mice on the control diet. Additionally, there was a TBI effect on macrophages at 1 DPI; TBI mice on the control diet had more macrophages than sham mice on the control diet (Fig. 1D).

There were no differences in macrophages between PLX and control mice at any time point. Sham mice on the PLX diet had more monocytes compared to sham mice on the control diet at both 1 and 7 DPI (Fig. 1E). There were no differences in monocytes between TBI and sham mice at any time point. Sham mice on the PLX diet had more neutrophils than sham mice on the control diet at 1 DPI (Fig. 1F). There was a PLX effect on TBI mice; TBI mice on the PLX diet had more neutrophils than TBI mice on the control diet at 7 DPI. There were no differences in TBI mice on the control diet compared to sham mice on the control diet, or TBI mice on the PLX diet compared to sham mice on the PLX diet, at any time point.

### Colony-stimulating factor-1 receptor inhibition altered monocyte populations and prevented traumatic brain injury–induced increases of peripheral myeloid cells and neutrophils

Immune cell populations were quantified using flow cytometry in blood (Fig. 2A). All posterior probabilities (*P*) and Bayes factors (*K*) are presented with their corresponding graphs of point estimates and confidence intervals in Figure 2. There were no differences in peripheral leukocytes among any groups at any time point (Fig. 2B). There was a TBI effect on myeloid cells in the periphery, regardless of diet, at 3 DPI; TBI resulted in a higher number of myeloid cells compared to shams for both control and PLX diets (Fig. 2C). There was a PLX effect on peripheral myeloid cells in TBI mice at 7 DPI; TBI mice on the PLX diet had more myeloid cells than sham mice on the control diet. There was a PLX effect, independent of injury, at 7 DPI, and the PLX diet resulted in fewer myeloid cells in sham mice compared to sham mice on the control diet. PLX had a substantial effect on CD115^+^ monocytes. All mice on the PLX diet, independent of TBI, had fewer monocytes compared to mice on the control diet, across all time points (Fig. 2D). There was also a TBI effect on monocytes; TBI mice on the control and PLX diets had more monocytes than their respective sham mice at 3 DPI. This elevation was also observed in TBI mice on the PLX diet compared to sham mice on the PLX diet at 1 DPI.

Peripheral neutrophils were elevated in TBI mice on the control diet compared to sham mice on the control diet at 1 DPI (Fig. 2E). Peripheral neutrophils were elevated in TBI mice on the PLX diet compared to sham mice on the PLX diet at 7 DPI. There was a PLX effect on peripheral neutrophils in TBI mice; TBI mice on the PLX diet had fewer neutrophils at 3 DPI and more neutrophils at 7 DPI compared to TBI mice on the control diet. Ly6C^high^ monocytes were elevated in both sham and TBI mice on the PLX diet compared to sham and TBI mice on the control diet at 3 and 7 DPI (Fig. 2F). Ly6C^int^ monocytes were elevated in both sham and TBI mice on the PLX diet compared to sham and TBI mice on the control diet at 1 and 7 DPI (Fig. 2G). At 3 DPI, there was a TBI effect, where TBI mice on the control diet had a higher number of Ly6C^int^ monocytes compared to sham mice on the control diet. Additionally, there was a PLX effect in sham, where sham mice on PLX had a higher number of Ly6C^int^ monocytes compared to sham control-diet mice.

There were fewer Ly6C^low^ monocytes in both sham and TBI mice on the PLX diet compared to sham and TBI mice on the control diet at 1 and 7 DPI (Fig. 2H). At 3 DPI, there was a TBI effect, where TBI mice on the control diet had fewer Ly6C^low^ monocytes compared to sham on the control diet, and a PLX effect in sham, where sham PLX had fewer Ly6C^low^ monocytes compared to the sham control diet. At 7 DPI, TBI mice on the PLX diet had fewer monocytes compared to sham mice on the PLX diet.

### Colony-stimulating factor-1 receptor inhibition in traumatic brain injury mice elevated proinflammatory cytokines and lowered anti-inflammatory cytokines in blood at 7 days post-injury

**FIG. 3.**
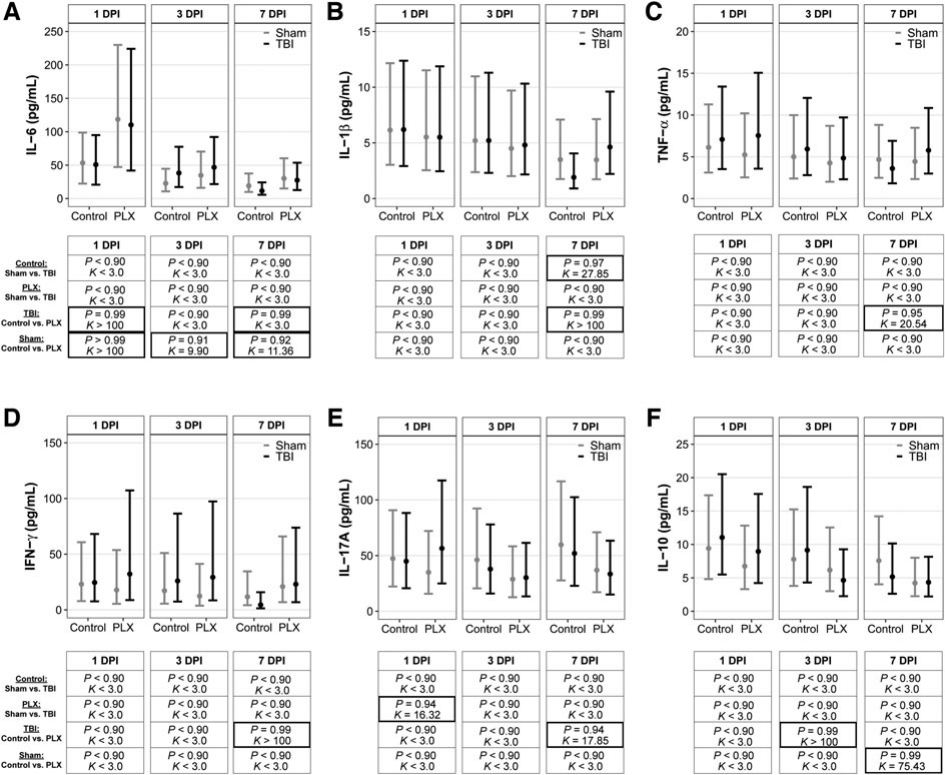
CSF-1R inhibition modulated peripheral cytokines after TBI. Peripheral pro- and anti-inflammatory cytokine levels in the blood for (**A**) interleukin (IL)-6, (**B**) IL-1β, (**C**) tumor necrosis factor (TNF)-α, (**D**) interferon (IFN)-γ, (**E**) IL-17A, and (**F**) IL-10. Results are presented graphically as marginal effects point estimates with 95% credible intervals along with tables of corresponding posterior probabilities (*P*) and Bayes factors (*K*). Statistically supported differences are indicated by bold black boxes. CSF-1R, colony-stimulating factor-1 receptor; DPI, days post-injury; PLX, PLX5622; TBI, traumatic brain injury.

Peripheral cytokines were quantified in blood samples (Fig. 3). All posterior probabilities (*P*) and Bayes factors (*K*) are presented with their corresponding graphs of point estimates and confidence intervals in Figure 3. There was a PLX effect on IL-6 independent of TBI. Sham mice on the PLX diet had elevated IL-6 compared to mice on the control diet at 1, 3, and 7 DPI (Fig. 3A). Additionally, there was a PLX × TBI, and TBI mice on the PLX diet had elevated IL-6 at 1 and 7 DPI compared to TBI mice on the control diet (Fig. 3A). TBI mice on the control diet had lower IL-1β levels compared to sham mice on the control diet at 3 DPI (Fig. 3B). There was a PLX effect in TBI mice, where TBI mice on the PLX diet had elevated IL-1β at 7 DPI compared to TBI mice on the control diet; there were no differences in IL-1β levels at 1 or 3 DPI among any groups. There were also no differences in TNF-α levels at 1 or 3 DPI among any groups (Fig. 3C). At 7 DPI, there was a PLX effect on TBI mice, where TBI PLX-diet mice had higher TNF-α levels compared to TBI mice on the control diet.

There were no differences in IFN-γ at 1 or 3 DPI among any groups (Fig. 3D). There was a PLX effect in TBI mice at 7 DPI, where TBI mice on the PLX diet had elevated IFN-γ compared to TBI mice on the control diet. At 1 DPI, TBI mice on the PLX diet had elevated IL-17A compared to sham mice on the PLX diet (Fig. 3E). There were no differences in IL-17A levels at 3 DPI. There was a PLX effect in TBI mice at 7 DPI, and TBI-PLX mice had lower IL-17A compared to TBI mice on the control diet. There were no differences in IL-10 among any groups at 1 DPI (Fig. 3F). At 3 DPI, there was a PLX effect in TBI mice and TBI PLX mice had lower IL-10 compared to TBI mice on the control diet. At 7 DPI, there was a PLX effect in sham mice, where sham mice on the PLX diet had lower IL-10 compared to sham mice on the control diet.

## Discussion

TBI initiates a robust neuroinflammatory response that is, in part, mediated by microglia. Microglia that remain reactive over a chronic period can establish or exacerbate disease progression; thus, pharmacological strategies to suppress the reactivity of microglia are being explored as therapies. Microglia have been targeted to improve functional outcome after acquired neurological injuries, but results have been conflicting. CSF-1R inhibition provided neuroprotection for intracerebral hemorrhage by preserving blood–brain barrier integrity and reducing leukocyte infiltration.^42^ Those findings are contrasted by studies showing that CSF-1R inhibition exacerbated neuroinflammation and brain pathology after ischemia.^43,44^ Microglia depleted with PLX before mFPI injury prevented TBI-associated functional and behavioral impairments in the mouse.^45^ However, PLX treatment before experimental stroke increased infarct volume and resulted in higher levels of infiltrating monocytes.^46^

Together, the pre-clinical literature suggests that targeting microglia before an acquired neurological injury could be either beneficial or detrimental to recovery. Elucidating the role of microglia in the central and peripheral immune response to TBI is critical to guide future studies that therapeutically target microglia to mitigate injury-induced deficits. Our study addresses a critical knowledge gap by investigating the microglial mechanisms in central and peripheral inflammation after experimental TBI.

We found that TBI elevated leukocytes, microglia, and macrophages in the brain. This central inflammatory response was altered by CSF-1R inhibition. TBI mice on the PLX diet did not have elevated levels of macrophages in the brain, and microglia and leukocyte levels were significantly lowered by CSF-1R inhibition before TBI. Interestingly, TBI mice on the PLX diet had elevated levels of neutrophils in the brain at 7 DPI. Neutrophils are early responders to tissue damage and one of the first cells to accumulate in the brain after TBI.^47^ Neutrophils can clear injury-induced debris by phagocytosis.^13^ However, neutrophils also release toxic molecules, including free radicals and proinflammatory cytokines, which can exacerbate tissue damage.^48^ Although TBI-induced inflammation resolved irrespective of intervention with PLX, CSF-1R inhibition sustained peripheral inflammation, which should be considered when using CSF-1R inhibition as a pre-treatment for TBI.

TBI also resulted in a robust peripheral immune response that peaked at 3 DPI. This peripheral response was characterized by elevated myeloid cells, neutrophils, and Ly6C^int^ monocytes in the blood. CSF-1R inhibition altered this peripheral response by lowering levels of peripheral myeloid cells and Ly6C^low^ monocytes, while elevating Ly6C^high^ and Ly6C^int^ monocytes. Ly6C^high^ monocytes are a short-lived inflammatory subset of monocytes that are actively recruited to sites of infection or injury.^49^ In contrast, Ly6C^low^ monocytes are patrolling resident monocytes that adhere to blood vessels under non-inflammatory conditions.^50^ Our findings suggest that CSF-1R inhibition, independent of TBI, redistributed peripheral monocyte populations toward an inflammatory state. We also observed a PLX × TBI interaction in the peripheral monocyte response. Ly6C^high^ monocyte populations were elevated, and Ly6C^low^ populations were reduced. This shift in monocyte populations at 7 DPI suggests a sustained peripheral response to TBI and supports that the monocyte response to TBI is, at least partially, mediated by microglia.

TBI-induced inflammation is communicated by pro- and anti-inflammatory cytokines, which may be below detectable levels in healthy tissue but rapidly increase in response to insults.^51,52^ Cytokines cause large inflammatory effects at small concentrations, but exhibit redundant functions that can lead to unregulated inflammation and progressive tissue damage after an injury.^2,53^ Interventions that target proinflammatory cytokines have been explored to treat symptoms associated with TBI and are a plausible target for therapeutics.^54^ We found a PLX × TBI interaction on peripheral cytokine levels after diffuse TBI. CSF-1R inhibition in brain-injured mice elevated peripheral proinflammatory cytokines, but lowered anti-inflammatory cytokines, at 7 DPI. This suggests that depleting microglia before experimental TBI may increase the peripheral inflammatory response. Similar results have been reported after the administration of lipopolysaccharide (LPS) in rats with depleted microglia.^55^ At 24 h post-injection, IL-1β and TNF-α were significantly elevated compared to vehicle-treated controls, suggesting that microglia depletion before an immune challenge exacerbates the proinflammatory response.^55^

In mice treated with PLX, CSF-1R inhibition blunted the IL-10 response to a peripheral inflammatory stimulus using LPS.^55^ In our study, IL-10 was lower in TBI-PLX mice at 7 DPI. Anti-inflammatory cytokines (e.g., IL-10) have been used to alter proinflammatory cytokine expression after TBI, and when administered after experimental TBI, IL-10 reduced TNF expression and improved neurological recovery in rats.^56^ The lower IL-10 levels observed in our study may be indicative of a prolonged inflammatory response in TBI mice on the PLX diet. Further studies are warranted to investigate the behavioral response and functional recovery after experimental TBI when CSF-1R has been inhibited before TBI.

We observed several PLX-dependent responses in the central and peripheral immune responses of shams, independent of TBI. PLX administration lowered levels of neutrophils in the brain, lowered levels of myeloid cells, neutrophils, and Ly6C^int^ monocytes in the blood, and elevated peripheral IL-6 levels at all time points. Importantly, a robust PLX effect on CD115^+^ peripheral monocytes was observed, with an almost complete elimination of circulating CD115^+^ monocytes after PLX administration. Collectively, these findings support off-target central and peripheral effects of microglia depletion by CSF-1R inhibition. Similar to our findings, microglia depletion with PLX3397 had significant effects on circulating monocytes and peripheral tissue macrophages,^57^ and PLX5622 administration resulted in long-term changes in bone-marrow–derived macrophages, in part, through suppression of IL-1β.^58^

We postulate that some off-target inflammatory effects of CSF-1R inhibition are a result of the clearance of dead microglia from the brain. The mechanism of eliminating and clearing microglial cells from the brain after PLX-induced depletion is not fully characterized, but there is evidence to support that microglia are eliminated through apoptosis.^43^ Microglia rapidly repopulate after the withdrawal of PLX, with measurable levels of microglia at 24 h post-PLX treatment, and full repopulation by 3 days.^59^

In our experimental TBI model, mice slept significantly more during the first 24 h post-injury,^60,61^ and subsequently eat and drink less, as noted in our post-operative evaluations that often require the administration of saline to brain-injured rodents to treat dehydration. It is possible that TBI suppressed the appetite of mice and therefore some microglia repopulated by 1 DPI, attributable to reduced oral consumption of PLX. We suspect that these rapidly repopulated microglia were depleted when mice resumed their normal feeding routine and were cleared by phagocytosis. Inflammatory cytokines play a role in phagocytosis and clearance of apoptotic cells and particles.^62,63^ Specifically, phagocytosis of apoptotic cellular debris induces inflammatory mediators, such IL-6, IL-1β, and TNF-α, that are released to enhance the activity of phagocytes.^62^ An increase in proinflammatory cytokines to prompt the phagocytosis of dead microglia may partially explain elevated proinflammatory levels in TBI mice on the PLX diet.

The results of this study should be interpreted in the context of some limitations. Though mice were monitored daily for food and water consumption, we did not directly measure food intake. After mFPI, it is possible that there was a transient decrease in food intake. This decrease in food consumption could have reduced the dose of PLX administered to TBI mice, which may have initiated the repopulation of microglia in the PLX groups. However, daily body weights were measured as part of our routine post-operative care, and no mouse lost >10% of their body weight. Therefore, it is unlikely that any mouse abstained from eating for a long enough period to initiate microglia repopulation.

We only used male mice, but evidence from our lab and others suggests that sex differences in response to TBI exist.^61,64,65^ Therefore, additional studies are needed to determine the effect of sex under the conditions similar to this study. Additionally, senescent microglia in the aging brain have an increased inflammatory profile at baseline and respond differently to TBI and other neurological insults.^66^ Depletion and repopulation of microglia in the aged brain through PLX administration and subsequent withdrawal did not fully reverse the aged-induced inflammatory profile of microglia,^67^ and therefore additional studies in aged mice are needed to better understand CSF-1R-mediated responses to TBI. We used CSF-1R inhibition as a pre-treatment for TBI, but substantial evidence supports that microglia depletion in the chronic phase of TBI can suppress neuroinflammatory pathways and improve neurological function.^9,16^

Pre-treatment with a pharmacological compound is not standard for persons at a high risk of sustaining TBI. In this study, microglia depletion pre-TBI was not used as a therapeutic strategy, but as a research tool to better understand how microglia contribute to the central and peripheral TBI-induced inflammatory response. Future studies will explore additional therapeutic windows, including delayed administration of PLX after TBI and the repopulation of microglia as a timed intervention.

## Conclusion

In conclusion, CSF-1R inhibition blunted the immune response to TBI at 1 and 3 DPI, but elevated peripheral inflammation at 7 DPI. CSF-1R inhibition may be a plausible mechanism to target both central and peripheral immune cells and change the inflammatory response to TBI. However, administration of PLX as a research tool to target microglia-mediated inflammation should be used with caution, given that we also observed off-target inflammatory effects of CSF-1R inhibition, independent of TBI. Specifically, CD115^+^ peripheral monocytes were depleted. Additional studies are needed to precisely regulate acute and chronic inflammation mediated by microglia to further elucidate the role of microglia in functional recovery and therapeutic development in the context of TBI.

## Supplementary Material

Supplemental data

Supplemental data

## Data Availability

The data sets supporting the conclusions of this article are publicly available in the Open Data Commons for Traumatic Brain Injury (ODC-TBI) at the following link: https://odc-tbi.org/data/769. Data will be made publicly available in the Open Data Commons for Traumatic Brain Injury (ODC-TBI) upon manuscript acceptance.

## Abbreviations Used

7-AAD: 7-aminoactinomycin D
CSF-1R: colony-stimulating factor-1 receptor
DPI: days post-injury
FACS: fluorescence-activated cell sorting
FPI: fluid percussion injury
IFN: interferon
IL: interleukin
LPS: lipopolysaccharide
mFPI: midline fluid percussion injury
PLX: PLX5622
RT: room temperature
TBI: traumatic brain injury
TNF: tumor necrosis factor
