# Supplementary material for: Colony-Stimulating Factor-1 Receptor Inhibition Transiently Attenuated the Peripheral Immune Response to Experimental Traumatic Brain Injury
Source: Neurotrauma Rep. 2023 Apr 28;4(1):284–96. doi: 10.1089/neur.2022.0092 (PMC10150725; doi:10.1089/neur.2022.0092)
Supplement: Supplemental data [file Suppl_TableS1.docx]

|  | **Live events** | **CD45^+^** | **CD11b^+^CD45^low^** | **CD11b^+^CD45^high^** | **CD45^+^Ly6C^+^Ly6G^-^** | **CD45^+^Ly6C^int^Ly6G^+^** |
| --- | --- | --- | --- | --- | --- | --- |
| **control sham**  **1 DPI** | 29576 ± 8977 | 3401 ± 2068 | 5449 ± 2796 | 1051 ± 624 | 321 ± 327 | 211 ± 178 |
| **control TBI**  **1 DPI** | 34205 ± 8443 | 4735 ± 2534 | 6566 ± 3267 | 2055 ± 1188 | 513 ± 339 | 482 ± 379 |
| **PLX sham**  **1 DPI** | 23066 ± 11388 | 1641 ± 1089 | 289 ± 270 | 751 ± 491 | 396 ± 521 | 318 ± 266 |
| **PLX TBI**  **1 DPI** | 27244 ± 9998 | 2030 ± 1487 | 896 ± 989 | 1043 ± 699 | 480 ± 567 | 232 ± 158 |
| **control sham**  **3 DPI** | 36007 ± 14864 | 1734 ± 750 | 7303 ± 3530 | 1061 ± 966 | 427 ± 425 | 226 ± 115 |
| **control TBI**  **3 DPI** | 37831 ± 9651 | 3812 ± 2615 | 9643 ± 3670 | 1440 ± 824 | 614 ± 484 | 619 ± 1006 |
| **PLX sham**  **3 DPI** | 29883 ± 12192 | 2646 ± 1918 | 1277 ± 940 | 989 ± 741 | 696 ± 1053 | 359 ± 231 |
| **PLX TBI**  **3 DPI** | 34288 ± 9388 | 2284 ± 1282 | 2018 ± 1797 | 851 ± 529 | 568 ± 399 | 339 ± 221 |
| **control sham**  **7 DPI** | 32284 ± 10827 | 4566 ± 3921 | 7306 ± 3137 | 1558 ± 1412 | 202 ± 131 | 280 ± 252 |
| **control TBI**  **7 DPI** | 35390 ± 9161 | 3282 ± 2264 | 7701 ± 3269 | 1196 ± 818 | 170 ± 215 | 131 ± 78 |
| **PLX sham**  **7 DPI** | 29310 ± 7439 | 2875 ± 1964 | 1187 ± 939 | 1311 ± 1241 | 344 ± 379 | 191 ± 97 |
| **PLX TBI**  **7 DPI** | 25955 ± 14075 | 1570 ± 783 | 800 ± 703 | 661 ± 362 | 174 ± 154 | 193 ± 132 |

**Supplemental Table 1: Brain flow cytometry events**

Data are presented as mean ± standard deviation
