# Supplementary material for: Colony-Stimulating Factor-1 Receptor Inhibition Transiently Attenuated the Peripheral Immune Response to Experimental Traumatic Brain Injury
Source: Neurotrauma Rep. 2023 Apr 28;4(1):284–96. doi: 10.1089/neur.2022.0092 (PMC10150725; doi:10.1089/neur.2022.0092)
Supplement: Supplemental data [file Suppl_TableS2.docx]

|  | **CD45^+^** | **CD45^+^CD11b^+^** | **CD45^+^CD11b^+^CD115^+^** | **CD45^+^CD11b^+^Ly6G^+^** | **CD45^+^CD11b^+^Ly6C^high^** | **CD45^+^CD11b^+^Ly6C^int^** | **CD45^+^CD11b^+^Ly6C^low^** |
| --- | --- | --- | --- | --- | --- | --- | --- |
| **control sham**  **1 DPI** | 91141 ± 4837 | 23667 ± 6727 | 4799 ± 1915 | 13799 ± 5928 | 2519 ± 935 | 17407 ± 5934 | 3224 ± 758 |
| **control TBI**  **1 DPI** | 91265 ± 7991 | 21250 ± 3117 | 4458 ± 1096 | 11191 ± 3115 | 2306 ± 744 | 15187 ± 2747 | 3556 ± 911 |
| **PLX sham**  **1 DPI** | 90159 ± 2924 | 24972 ± 8699 | 3654 ± 2240 | 16397 ± 7866 | 2002 ± 885 | 19569 ± 7638 | 2729 ± 1081 |
| **PLX TBI**  **1 DPI** | 90472 ± 4494 | 24198 ± 4660 | 2714 ± 2072 | 16259 ± 5290 | 2149 ± 721 | 18961 ± 5038 | 2528 ± 697 |
| **control sham**  **3 DPI** | 85438 ± 12625 | 25400 ± 12890 | 3558 ± 2089 | 16524 ± 13499 | 2469 ± 1351 | 20291 ± 13401 | 2589 ± 836 |
| **control TBI**  **3 DPI** | 88157 ± 3737 | 23656 ± 7746 | 5814 ± 2496 | 13384 ± 8451 | 2655 ± 1214 | 16986 ± 8429 | 3943 ± 1870 |
| **PLX sham**  **3 DPI** | 95466 ± 8424 | 22137 ± 4784 | 4218 ± 2821 | 9948 ± 2977 | 3057 ± 774 | 14977 ± 3630 | 4003 ± 1370 |
| **PLX TBI**  **3 DPI** | 96445 ± 16682 | 20707 ± 6903 | 4050 ± 4038 | 9542 ± 3362 | 2980 ± 992 | 14304 ± 5115 | 3348 ± 2269 |
| **control sham**  **7 DPI** | 93764 ± 10302 | 22528 ± 4660 | 4584 ± 2535 | 10631 ± 2449 | 2962 ± 1256 | 15584 ± 2949 | 3903 ± 2118 |
| **control TBI**  **7 DPI** | 91519 ± 4268 | 21857 ± 3298 | 3935 ± 2574 | 10984 ± 2504 | 3129 ± 772 | 15693 ± 3031 | 2969 ± 935 |
| **PLX sham**  **7 DPI** | 94183 ± 4849 | 25906 ± 7617 | 6364 ± 3113 | 13246 ± 5007 | 3279 ± 1278 | 17950 ± 5275 | 4582 ± 2395 |
| **PLX TBI**  **7 DPI** | 89979 ± 4010 | 26133 ± 6568 | 5153 ± 3698 | 13462 ± 6zoom095 | 3885 ± 1606 | 18534 ± 6186 | 3620 ± 1821 |

**Supplemental Table 2: Blood flow cytometry events**

Data are presented as mean ± standard deviation
